# Poly[tetra­aqua­(5-hy­droxy­pyridin-1-ium-3-carboxyl­ato-κ*O* ^3^)tris­(μ-oxalato-κ^4^ *O* ^1^,*O* ^2^:*O* ^1′^,*O* ^2′^)dieuropium(III)]

**DOI:** 10.1107/S1600536813011057

**Published:** 2013-04-27

**Authors:** Shan-Shan Xu, Jun-Long Mi, Hong-Ji Chen

**Affiliations:** a Department of Materials Science and Engineering, Jinan University, Guangzhou 510632, People’s Republic of China

## Abstract

In the title compound, [Eu_2_(C_6_H_5_NO_3_)_2_(C_2_O_4_)_3_(H_2_O)_4_]_*n*_, the Eu^III^ atom is bonded to one O atom from a monodentate 5-hy­droxy­pyridin-1-ium-3-carboxyl­ate ligand, six O atoms from three oxalate ligands and two water mol­ecules, exhibiting a highly distorted tricapped trigonal geometry. Three independent oxalate ligands, each lying on an inversion center, bridge the Eu^III^ atoms, forming a brickwall-like layer parallel to (001), which is stabilized by intra­layer O—H⋯O hydrogen bonds. The layers are further linked through inter­layer O—H⋯O and N—H⋯O hydrogen bonds and π–π inter­actions between the pyridine rings [centroid–centroid distance = 3.5741 (14) Å] into a three-dimensional supra­molecular network.

## Related literature

For background to metal complexes of pyridine-carb­oxy­lic derivatives, see: Black *et al.* (2009 ▶); Cañadillas-Delgado *et al.* (2010 ▶); Hu *et al.* (2007 ▶); Sun *et al.* (2010 ▶); Wen *et al.* (2007 ▶); Xu *et al.* (2008 ▶). For structures and properties of coordination polymers derived from 5-hy­droxy­nicotinic acid, see: Bunzli (2010 ▶); Decadt *et al.* (2012 ▶); Gai *et al.* (2012 ▶); Ramya *et al.* (2012 ▶); Yang *et al.* (2011 ▶); Zhang *et al.* (2012 ▶).
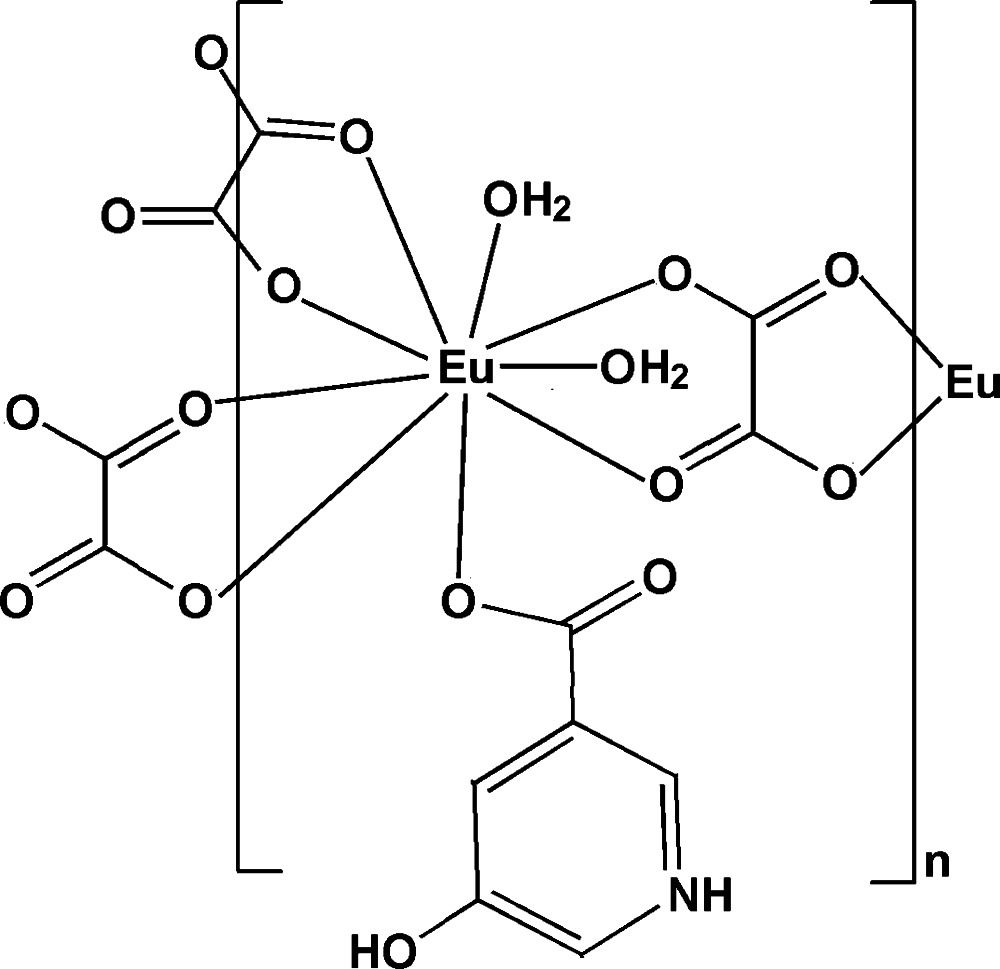


## Experimental

### 

#### Crystal data

[Eu_2_(C_6_H_5_NO_3_)_2_(C_2_O_4_)_3_(H_2_O)_4_]
*M* *_r_* = 918.26
Triclinic, 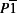

*a* = 7.5912 (2) Å
*b* = 8.0973 (3) Å
*c* = 10.6706 (3) Å
α = 103.493 (3)°
β = 98.589 (3)°
γ = 92.240 (3)°
*V* = 628.78 (3) Å^3^
*Z* = 1
Mo *K*α radiation
μ = 5.05 mm^−1^
*T* = 153 K
0.24 × 0.17 × 0.08 mm

#### Data collection

Bruker APEXII CCD diffractometer
Absorption correction: multi-scan (*SADABS*; Sheldrick, 1996 ▶) *T*_min_ = 0.377, *T*_max_ = 0.688
12455 measured reflections
3135 independent reflections
2965 reflections with *I* > 2σ(*I*)
*R*_int_ = 0.037

#### Refinement

*R*[*F* ^2^ > 2σ(*F* ^2^)] = 0.018
*wR*(*F* ^2^) = 0.038
*S* = 1.09
3135 reflections
221 parameters
H atoms treated by a mixture of independent and constrained refinement
Δρ_max_ = 0.55 e Å^−3^
Δρ_min_ = −0.54 e Å^−3^

### 

Data collection: *APEX2* (Bruker, 2007 ▶); cell refinement: *SAINT* (Bruker, 2007 ▶); data reduction: *SAINT*; program(s) used to solve structure: *SHELXTL* (Sheldrick, 2008 ▶); program(s) used to refine structure: *SHELXTL*; molecular graphics: *DIAMOND* (Brandenburg, 1999 ▶); software used to prepare material for publication: *SHELXTL*.

## Supplementary Material

Crystal structure: contains datablock(s) I, global. DOI: 10.1107/S1600536813011057/hy2623sup1.cif

Structure factors: contains datablock(s) I. DOI: 10.1107/S1600536813011057/hy2623Isup2.hkl

Additional supplementary materials:  crystallographic information; 3D view; checkCIF report

## Figures and Tables

**Table 1:** Hydrogen-bond geometry (Å, °)

*D*—H⋯*A*	*D*—H	H⋯*A*	*D*⋯*A*	*D*—H⋯*A*
N1—H1⋯O9^i^	0.83 (3)	1.84 (3)	2.662 (3)	172 (3)
O3—H3*A*⋯O2^ii^	0.82	1.73	2.543 (2)	169
O10—H7⋯O4^iii^	0.76 (4)	2.26 (4)	3.016 (2)	168 (4)
O10—H8⋯O2^iv^	0.92 (4)	1.85 (4)	2.759 (3)	170 (3)
O11—H9⋯O6^iii^	0.88 (4)	1.90 (4)	2.771 (3)	170 (4)
O11—H10⋯O3^v^	0.74 (4)	2.04 (4)	2.776 (3)	172 (4)

Symmetry codes: (i) 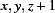; (ii) 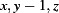; (iii) 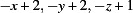; (iv) 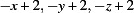; (v) 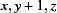.

**Fig. 1.**
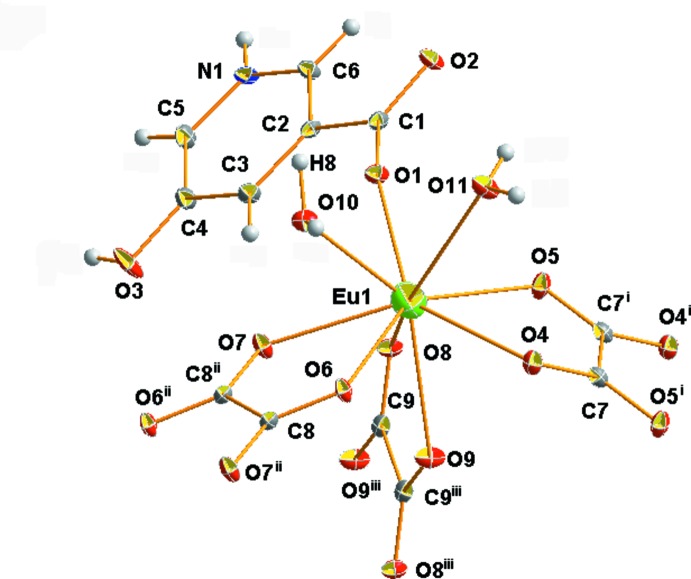
The asymmetric unit of the title compound, showing displacement ellipsoids at the 50% probability level. [Symmetry codes: (i) -x+1, -y+2, -z+1; (ii) -x+2, -y+1, -z+1; (iii) -x+1, -y+1, -z+1.]

**Fig. 2.**
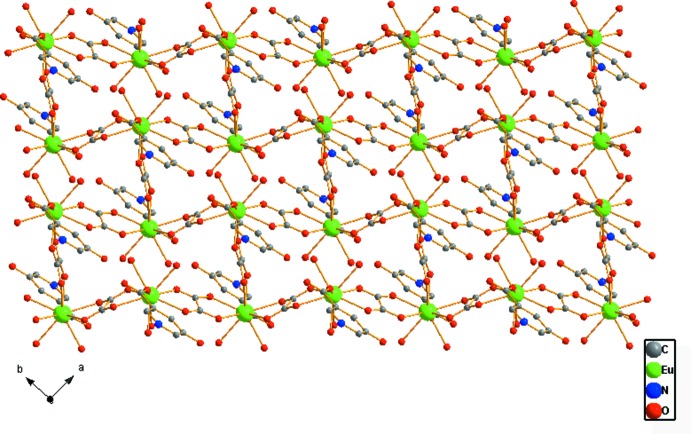
The layer structure of the title compound parallel to (001). H atoms are omitted for clarity.

## supplementary crystallographic information

### Comment

Pyridine-carboxylic derivatives are of resent interest in coordination polymers due to their structural diverity and unique physical properties (Black *et al.*, 2009; Cañadillas-Delgado *et al.*, 2010). Some transition and/or rare earth metal complexes with pyridine-2,5/6-dicarboxylic acid (Sun *et al.*, 2010; Wen *et al.*, 2007) or 2/6-hydroxynicotinic acid (Hu *et al.*, 2007; Xu *et al.*, 2008) have been prepared and documented. Recently, a few coordination polymers from 5-hydroxynicotinic acid are reported, in which the multidentate bridging ligand exhibits versatile coordination modes in constructing transition metal (Yang *et al.*, 2011) and rare earth metal (Zhang *et al.*, 2012) organic frameworks. The research interest in europium(III) coordination polymers with the ligands comes from their luminescent properties (Decadt *et al.*, 2012; Gai *et al.*, 2012; Ramya *et al.*, 2012) and applications (Bunzli, 2010). Here, we report the crystal structure of an europium(III) complex of a pyridine-carboxylic derivative.

The title compound is isostructural with its Tb(III) and Sm(III) analogues (Zhang *et al.*, 2012). As shown in Fig. 1, the asymmetric unit contains an Eu^III^ ion, a 3-*H*-5-hydroxynicotinate ligand, one and half oxalate ligands and two coordinated water molecules. The Eu^III^ ion is bonded to nine O atoms, one from the 3-*H*-5-hydroxynicotinate ligand, six from three oxalate ligands and two from the water molecules, exhibiting a highly distorted tricapped trigonal geometry. The Eu^III^ ions are linked by the oxalate ligands into a brickwall-like layer parallel to (001) (Fig. 2), with Eu···Eu distances of 6.1886 (3), 6.2845 (3) and 6.4206 (3) Å. In the compound, the three oxalate ligands are centrosymmetric and bridge metal ions in a side-by-side coordination manner. The layers are stabilized by intralayer O—H···O hydrogen bonds and further linked through interlayer O—H···O hydrogen bonds and π–π interactions between the pyridine rings [centroid–centroid distance = 3.5741 (14) Å] into a three-dimensional supramolecular network.

### Experimental

A mixture of europium nitrate (0.4 mmol, 0.186 g), 5-hydroxynicotinic acid (0.8 mmol, 0.112 g), ammonium oxalate (0.8 mmol, 0.099 g) and 10 ml water was sealed in a 15 ml Teflon-lined autoclave. Colorless crystals suitable for X-ray analysis were obtained by heating the mixture at 443 K for 70 h and then cooled down to room temperature at a rate of 5 K/h (yield: 45%). Analysis, calculated for C_9_H_9_EuNO_11_: C 23.54, H 1.98, N 3.05%; found: C 23.36, H 2.02, N 3.01%.

### Refinement

C-bound H atoms and H atom on hydroxyl were positioned geometrically and refined as riding atoms, with C—H = 0.93 and O—H = 0.82 Å and with *U*_iso_(H) = 1.2(1.5 for hydroxyl)*U*_eq_(C,O). Other H atoms were located from a difference Fourier map and refined isotropically.

### Crystal data

**Table:**

[Eu_2_(C_6_H_5_NO_3_)_2_(C_2_O_4_)_3_(H_2_O)_4_]	*Z* = 1
*M**_r_* = 918.26	*F*(000) = 442
Triclinic, *P*1	*D*_x_ = 2.425 Mg m^−^^3^
Hall symbol: -P 1	Mo *K*α radiation, λ = 0.71073 Å
*a* = 7.5912 (2) Å	Cell parameters from 3135 reflections
*b* = 8.0973 (3) Å	θ = 2.6–29.4°
*c* = 10.6706 (3) Å	µ = 5.05 mm^−^^1^
α = 103.493 (3)°	*T* = 153 K
β = 98.589 (3)°	Block, colorless
γ = 92.240 (3)°	0.24 × 0.17 × 0.08 mm
*V* = 628.78 (3) Å^3^	

### Data collection

**Table:**

Bruker APEXII CCD diffractometer	3135 independent reflections
Radiation source: fine-focus sealed tube	2965 reflections with *I* > 2σ(*I*)
Graphite monochromator	*R*_int_ = 0.037
φ and ω scans	θ_max_ = 29.5°, θ_min_ = 2.6°
Absorption correction: multi-scan (*SADABS*; Sheldrick, 1996)	*h* = −9→9
*T*_min_ = 0.377, *T*_max_ = 0.688	*k* = −11→10
12455 measured reflections	*l* = −13→14

### Refinement

**Table:**

Refinement on *F*^2^	Secondary atom site location: difference Fourier map
Least-squares matrix: full	Hydrogen site location: inferred from neighbouring sites
*R*[*F*^2^ > 2σ(*F*^2^)] = 0.018	H atoms treated by a mixture of independent and constrained refinement
*wR*(*F*^2^) = 0.038	*w* = 1/[σ^2^(*F*_o_^2^) + (0.0087*P*)^2^ + 0.3669*P*] where *P* = (*F*_o_^2^ + 2*F*_c_^2^)/3
*S* = 1.09	(Δ/σ)_max_ = 0.002
3135 reflections	Δρ_max_ = 0.55 e Å^−^^3^
221 parameters	Δρ_min_ = −0.54 e Å^−^^3^
0 restraints	Extinction correction: *SHELXL*, Fc^*^=kFc[1+0.001xFc^2^λ^3^/sin(2θ)]^-1/4^
Primary atom site location: structure-invariant direct methods	Extinction coefficient: 0.0094 (4)

### Special details

**Table:**

Experimental. IR (cm^-1^, KBr): 3495(s), 3380(s), 3103(m), 3076(w), 3047(w), 2929(w), 2461(m), 2146(m), 1968(w), 1893(w), 1695(s), 1655(s), 1621(s), 1602(s), 1575(s), 1379(s), 1320(s), 1256(m), 1147(w), 1114(w), 1010(w), 935(w), 880(m), 806(s), 784(s), 667(m), 620(w), 565(w), 531(w), 484(m).
Geometry. All e.s.d.'s (except the e.s.d. in the dihedral angle between two l.s. planes) are estimated using the full covariance matrix. The cell e.s.d.'s are taken into account individually in the estimation of e.s.d.'s in distances, angles and torsion angles; correlations between e.s.d.'s in cell parameters are only used when they are defined by crystal symmetry. An approximate (isotropic) treatment of cell e.s.d.'s is used for estimating e.s.d.'s involving l.s. planes.
Refinement. Refinement of *F*^2^ against ALL reflections. The weighted *R*-factor *wR* and goodness of fit *S* are based on *F*^2^, conventional *R*-factors *R* are based on *F*, with *F* set to zero for negative *F*^2^. The threshold expression of *F*^2^ > σ(*F*^2^) is used only for calculating *R*-factors(gt) *etc*. and is not relevant to the choice of reflections for refinement. *R*-factors based on *F*^2^ are statistically about twice as large as those based on *F*, and *R*- factors based on ALL data will be even larger.

### Fractional atomic coordinates and isotropic or equivalent isotropic displacement parameters (Å^2^)

**Table:**

	*x*	*y*	*z*	*U*_iso_*/*U*_eq_	
C1	0.7687 (3)	0.8846 (3)	0.9610 (2)	0.0095 (5)	
C2	0.7420 (3)	0.7295 (3)	1.0146 (2)	0.0094 (5)	
C3	0.7733 (3)	0.5691 (3)	0.9454 (2)	0.0116 (5)	
H3	0.8082	0.5552	0.8639	0.014*	
C4	0.7525 (4)	0.4283 (3)	0.9978 (2)	0.0127 (5)	
C5	0.7046 (4)	0.4541 (3)	1.1205 (2)	0.0132 (5)	
H5	0.6934	0.3631	1.1590	0.016*	
C6	0.6887 (3)	0.7479 (3)	1.1353 (2)	0.0110 (5)	
H6	0.6631	0.8540	1.1818	0.013*	
C7	0.5536 (3)	1.0035 (3)	0.4431 (2)	0.0099 (5)	
C8	1.0255 (3)	0.5479 (3)	0.4489 (2)	0.0084 (5)	
C9	0.4854 (3)	0.4971 (3)	0.5697 (2)	0.0101 (5)	
Eu1	0.797021 (16)	0.810935 (14)	0.626500 (11)	0.00700 (5)	
N1	0.6745 (3)	0.6119 (3)	1.1836 (2)	0.0112 (4)	
O1	0.8197 (2)	0.8624 (2)	0.85299 (16)	0.0118 (4)	
O2	0.7395 (3)	1.0258 (2)	1.03073 (16)	0.0141 (4)	
O3	0.7803 (3)	0.2743 (2)	0.92694 (18)	0.0225 (5)	
H3A	0.7713	0.2035	0.9698	0.034*	
O4	0.7120 (2)	0.9644 (2)	0.45635 (16)	0.0117 (4)	
O5	0.5274 (2)	0.9519 (2)	0.65247 (17)	0.0138 (4)	
O6	0.9752 (2)	0.6961 (2)	0.45804 (16)	0.0103 (3)	
O7	0.8889 (2)	0.5283 (2)	0.63432 (17)	0.0120 (4)	
O8	0.5549 (2)	0.6147 (2)	0.66268 (16)	0.0117 (4)	
O9	0.6111 (2)	0.6314 (2)	0.42511 (16)	0.0138 (4)	
O10	1.1269 (2)	0.8348 (2)	0.70926 (19)	0.0138 (4)	
O11	0.9123 (3)	1.1139 (2)	0.7043 (2)	0.0161 (4)	
H1	0.645 (4)	0.622 (4)	1.257 (3)	0.025 (9)*	
H7	1.179 (5)	0.876 (4)	0.667 (4)	0.032 (11)*	
H8	1.168 (5)	0.893 (4)	0.794 (4)	0.038 (10)*	
H9	0.937 (5)	1.181 (5)	0.654 (4)	0.050 (12)*	
H10	0.874 (5)	1.164 (4)	0.760 (4)	0.027 (10)*	

### Atomic displacement parameters (Å^2^)

**Table:**

	*U*^11^	*U*^22^	*U*^33^	*U*^12^	*U*^13^	*U*^23^
C1	0.0109 (12)	0.0101 (11)	0.0078 (12)	−0.0005 (9)	0.0008 (9)	0.0036 (9)
C2	0.0096 (12)	0.0113 (11)	0.0076 (12)	−0.0008 (9)	0.0007 (9)	0.0037 (9)
C3	0.0162 (13)	0.0124 (12)	0.0073 (12)	0.0010 (10)	0.0041 (9)	0.0035 (9)
C4	0.0177 (13)	0.0096 (12)	0.0118 (13)	0.0024 (10)	0.0037 (10)	0.0034 (9)
C5	0.0191 (14)	0.0104 (12)	0.0127 (13)	0.0025 (10)	0.0051 (10)	0.0060 (9)
C6	0.0151 (13)	0.0080 (11)	0.0101 (12)	−0.0007 (10)	0.0036 (9)	0.0015 (9)
C7	0.0127 (12)	0.0076 (11)	0.0106 (12)	0.0009 (9)	0.0053 (9)	0.0025 (9)
C8	0.0074 (12)	0.0105 (11)	0.0078 (11)	0.0007 (9)	0.0009 (9)	0.0030 (9)
C9	0.0080 (12)	0.0138 (12)	0.0103 (13)	0.0028 (10)	0.0037 (9)	0.0045 (9)
Eu1	0.00918 (8)	0.00665 (7)	0.00609 (7)	0.00141 (4)	0.00295 (4)	0.00217 (4)
N1	0.0159 (11)	0.0124 (10)	0.0056 (10)	−0.0002 (9)	0.0039 (8)	0.0014 (8)
O1	0.0162 (9)	0.0119 (8)	0.0077 (9)	−0.0008 (7)	0.0031 (7)	0.0027 (6)
O2	0.0256 (10)	0.0077 (8)	0.0102 (9)	0.0014 (7)	0.0067 (7)	0.0019 (7)
O3	0.0487 (14)	0.0073 (9)	0.0173 (10)	0.0068 (9)	0.0196 (9)	0.0049 (7)
O4	0.0101 (9)	0.0144 (9)	0.0126 (9)	0.0033 (7)	0.0047 (7)	0.0052 (7)
O5	0.0154 (9)	0.0194 (9)	0.0107 (9)	0.0078 (7)	0.0051 (7)	0.0088 (7)
O6	0.0128 (9)	0.0080 (8)	0.0129 (9)	0.0032 (7)	0.0067 (7)	0.0049 (6)
O7	0.0170 (9)	0.0095 (8)	0.0131 (9)	0.0045 (7)	0.0096 (7)	0.0047 (7)
O8	0.0125 (9)	0.0142 (9)	0.0082 (9)	−0.0020 (7)	0.0027 (7)	0.0020 (7)
O9	0.0173 (10)	0.0148 (9)	0.0098 (9)	−0.0066 (7)	0.0049 (7)	0.0038 (7)
O10	0.0118 (9)	0.0165 (9)	0.0123 (10)	−0.0015 (8)	0.0031 (7)	0.0016 (8)
O11	0.0274 (12)	0.0109 (9)	0.0122 (10)	0.0002 (8)	0.0090 (8)	0.0035 (8)

### Geometric parameters (Å, º)

**Table:**

C1—O1	1.246 (3)	C9—O9^iii^	1.266 (3)
C1—O2	1.258 (3)	C9—C9^iii^	1.546 (5)
C1—C2	1.515 (3)	Eu1—O1	2.3333 (16)
C2—C6	1.384 (3)	Eu1—O5	2.4025 (18)
C2—C3	1.385 (3)	Eu1—O7	2.4348 (16)
C3—C4	1.395 (3)	Eu1—O6	2.4433 (17)
C3—H3	0.9300	Eu1—O4	2.4539 (17)
C4—O3	1.341 (3)	Eu1—O11	2.4776 (18)
C4—C5	1.383 (3)	Eu1—O9	2.4899 (17)
C5—N1	1.344 (3)	Eu1—O10	2.5118 (19)
C5—H5	0.9300	Eu1—O8	2.5142 (16)
C6—N1	1.328 (3)	N1—H1	0.83 (3)
C6—H6	0.9300	O3—H3A	0.8200
C7—O5^i^	1.246 (3)	O5—C7^i^	1.246 (3)
C7—O4	1.252 (3)	O7—C8^ii^	1.241 (3)
C7—C7^i^	1.569 (5)	O9—C9^iii^	1.266 (3)
C8—O7^ii^	1.241 (3)	O10—H7	0.76 (4)
C8—O6	1.260 (3)	O10—H8	0.92 (4)
C8—C8^ii^	1.562 (5)	O11—H9	0.89 (4)
C9—O8	1.238 (3)	O11—H10	0.74 (4)
			
O1—C1—O2	125.5 (2)	O1—Eu1—O8	75.12 (6)
O1—C1—C2	117.7 (2)	O5—Eu1—O8	68.60 (6)
O2—C1—C2	116.8 (2)	O7—Eu1—O8	66.05 (6)
C6—C2—C3	119.3 (2)	O6—Eu1—O8	117.53 (5)
C6—C2—C1	119.8 (2)	O4—Eu1—O8	115.81 (6)
C3—C2—C1	120.9 (2)	O11—Eu1—O8	138.86 (6)
C2—C3—C4	120.1 (2)	O9—Eu1—O8	64.36 (5)
C2—C3—H3	119.9	O10—Eu1—O8	129.19 (6)
C4—C3—H3	119.9	O1—Eu1—C7^i^	100.78 (6)
O3—C4—C5	123.0 (2)	O5—Eu1—C7^i^	20.20 (6)
O3—C4—C3	118.5 (2)	O7—Eu1—C7^i^	140.16 (6)
C5—C4—C3	118.5 (2)	O6—Eu1—C7^i^	120.37 (6)
N1—C5—C4	119.3 (2)	O4—Eu1—C7^i^	48.67 (6)
N1—C5—H5	120.4	O11—Eu1—C7^i^	78.99 (6)
C4—C5—H5	120.4	O9—Eu1—C7^i^	70.91 (6)
N1—C6—C2	119.0 (2)	O10—Eu1—C7^i^	148.31 (6)
N1—C6—H6	120.5	O8—Eu1—C7^i^	77.75 (6)
C2—C6—H6	120.5	O1—Eu1—C7	125.61 (6)
O5^i^—C7—O4	125.9 (2)	O5—Eu1—C7	48.20 (6)
O5^i^—C7—C7^i^	116.6 (3)	O7—Eu1—C7	141.25 (6)
O4—C7—C7^i^	117.5 (3)	O6—Eu1—C7	93.32 (6)
O7^ii^—C8—O6	125.7 (2)	O4—Eu1—C7	20.65 (6)
O7^ii^—C8—C8^ii^	117.4 (3)	O11—Eu1—C7	75.30 (6)
O6—C8—C8^ii^	116.9 (3)	O9—Eu1—C7	62.82 (6)
O8—C9—O9^iii^	126.9 (2)	O10—Eu1—C7	132.68 (6)
O8—C9—C9^iii^	118.7 (3)	O8—Eu1—C7	98.11 (6)
O9^iii^—C9—C9^iii^	114.4 (3)	C7^i^—Eu1—C7	28.45 (8)
O1—Eu1—O5	80.96 (6)	O1—Eu1—C8^ii^	102.84 (6)
O1—Eu1—O7	85.97 (6)	O5—Eu1—C8^ii^	146.68 (6)
O5—Eu1—O7	134.62 (6)	O7—Eu1—C8^ii^	19.51 (6)
O1—Eu1—O6	138.32 (6)	O6—Eu1—C8^ii^	47.81 (6)
O5—Eu1—O6	140.49 (6)	O4—Eu1—C8^ii^	119.19 (6)
O7—Eu1—O6	67.29 (5)	O11—Eu1—C8^ii^	135.25 (6)
O1—Eu1—O4	137.52 (6)	O9—Eu1—C8^ii^	71.90 (6)
O5—Eu1—O4	67.69 (6)	O10—Eu1—C8^ii^	67.29 (6)
O7—Eu1—O4	136.49 (6)	O8—Eu1—C8^ii^	80.29 (6)
O6—Eu1—O4	75.83 (6)	C7^i^—Eu1—C8^ii^	142.17 (6)
O1—Eu1—O11	76.53 (6)	C7—Eu1—C8^ii^	129.65 (6)
O5—Eu1—O11	78.03 (7)	C6—N1—C5	123.8 (2)
O7—Eu1—O11	140.07 (7)	C6—N1—H1	120 (2)
O6—Eu1—O11	103.48 (6)	C5—N1—H1	116 (2)
O4—Eu1—O11	69.67 (6)	C1—O1—Eu1	158.00 (17)
O1—Eu1—O9	139.48 (6)	C4—O3—H3A	109.5
O5—Eu1—O9	83.77 (6)	C7—O4—Eu1	115.60 (15)
O7—Eu1—O9	78.60 (6)	C7^i^—O5—Eu1	118.04 (15)
O6—Eu1—O9	67.69 (6)	C8—O6—Eu1	118.79 (14)
O4—Eu1—O9	66.30 (6)	C8^ii^—O7—Eu1	119.54 (15)
O11—Eu1—O9	135.93 (6)	C9—O8—Eu1	118.26 (15)
O1—Eu1—O10	75.35 (6)	C9^iii^—O9—Eu1	120.53 (15)
O5—Eu1—O10	143.27 (6)	Eu1—O10—H7	111 (3)
O7—Eu1—O10	71.49 (6)	Eu1—O10—H8	119 (2)
O6—Eu1—O10	66.30 (6)	H7—O10—H8	105 (3)
O4—Eu1—O10	113.98 (6)	Eu1—O11—H9	125 (3)
O11—Eu1—O10	69.47 (7)	Eu1—O11—H10	116 (3)
O9—Eu1—O10	131.87 (6)	H9—O11—H10	109 (4)

Symmetry codes: (i) −*x*+1, −*y*+2, −*z*+1; (ii) −*x*+2, −*y*+1, −*z*+1; (iii) −*x*+1, −*y*+1, −*z*+1.

### Hydrogen-bond geometry (Å, º)

**Table:**

*D*—H···*A*	*D*—H	H···*A*	*D*···*A*	*D*—H···*A*
N1—H1···O9^iv^	0.83 (3)	1.84 (3)	2.662 (3)	172 (3)
O3—H3*A*···O2^v^	0.82	1.73	2.543 (2)	169
O10—H7···O4^vi^	0.76 (4)	2.26 (4)	3.016 (2)	168 (4)
O10—H8···O2^vii^	0.92 (4)	1.85 (4)	2.759 (3)	170 (3)
O11—H9···O6^vi^	0.88 (4)	1.90 (4)	2.771 (3)	170 (4)
O11—H10···O3^viii^	0.74 (4)	2.04 (4)	2.776 (3)	172 (4)

Symmetry codes: (iv) *x*, *y*, *z*+1; (v) *x*, *y*−1, *z*; (vi) −*x*+2, −*y*+2, −*z*+1; (vii) −*x*+2, −*y*+2, −*z*+2; (viii) *x*, *y*+1, *z*.
